# Ocular Syphilis Mimicking Giant Cell Arteritis

**DOI:** 10.7759/cureus.24715

**Published:** 2022-05-04

**Authors:** Areebah Qadir, Aemen S Khakwani, Mohammad R Khan, Nabiha Mustafa, Duaa Javaid, Sarah Siddiqui, Subhan Shah, Usman A Khan

**Affiliations:** 1 Internal Medicine, Bakhtawar Amin Medical and Dental College, Multan, PAK; 2 Internal Medicine, Suburban Community Hospital, Norriton East, USA; 3 Microbiology, Dr. Bhimrao Ambedkar University, Agra, IND; 4 Internal Medicine, Aston University, Birmingham, GBR; 5 Internal Medicine and Nephrology, University of Oklahoma Health Sciences Center, Oklahoma City, USA

**Keywords:** headache, visual loss, autoimmune, giant cell arteritis, ocular syphilis, syphilis

## Abstract

Syphilis is a rare cause of vision loss that mostly occurs after an infection of the meninges, brain tissue, and parenchyma. Syphilis can mimic auto-immune disease like giant cell arteritis which also manifest as sudden vision loss. Spirochete *Treponema pallidum* can spread through sexual contact and cause painless ulcers. Spirochetes can disseminate systemically and lead to secondary syphilis. Ocular syphilis can affect all parts of the eye in secondary and tertiary stages. It can present as scleritis, inflammation of the optic nerve, and uveitis.

We present the case of a 59- year-old male suffering from severe vision loss in the left eye and headache initially misdiagnosed with giant cell arteritis. He was correctly diagnosed with ocular syphilis after seeing a red macular rash on palms and soles, and was given penicillin G and probenecid. His visual acuity and field of vision improved soon.

Ocular syphilis is usually diagnosed late or misdiagnosed and leads to irreversible vision loss. Physicians should keep in mind the possibility of ocular syphilis in patients presenting with a sudden loss of vision and severe headaches.

## Introduction

Annually, there are an estimated six million new cases of syphilis globally in patients aged 15 to 49 years old [1]. Syphilis is a chronic sexually-transmitted disease caused by a bacterial infection by *Treponema pallium*. The infecting organism is difficult to cultivate in vitro and so there has been limited research regarding its biological basis and there is currently no vaccine, although it is responsive to penicillin treatment. However, it is still highly infectious and can survive for decades in the untreated host [2]. The hematogenous dissemination of treponemes from syphilitic ulcers results in rapid systemic infection, bringing on an early invasion of distant tissues including the central nervous system. This may then lead to neurosyphilis, ocular syphilis, or otic syphilis. Neurologic symptoms, including those of ocular syphilis, bear no pathognomonic characteristics and thus are often overlooked [3]. Due to the wide variety of clinical manifestations of ocular syphilis, the differential diagnoses vary significantly. Causes may be infectious, autoimmune, traumatic, or vascular [4]. One such vascular cause of similar symptoms is giant cell arteritis (GCA) which like ocular syphilis, classically manifests with vision loss arising due to ischemic optic neuropathy [5]. Diagnosis of syphilis might overlap with that of GCA as both present sudden vision loss and worsening headaches. But the point of demarcation between both is posterior uveitis and iritis taking place only in the syphilitic patient. Dilated iris vessels aka iris roseola and elevated intraocular pressure are relatively specific for ocular syphilis as well [6]. The following case report investigated the presentation of acute left vision loss in a 59-years-old homosexual male, who was initially treated as giant cell arteritis and was later found to be having ocular syphilis with posterior uveitis.

## Case presentation

A 59-year-old patient with asthma, anxiety, and past history of prostate cancer, presenting with worsening headaches, weakness, and graying out of the vision for the past two months was seen by rheumatology. He was empirically started on prednisone and referred for temporal artery biopsy. The patient’s headaches and vision problems were seen to improve upon the use of steroids. After two weeks the patient underwent a temporal artery biopsy which came back negative for arteritis, so the prednisone was tapered off. 

The patient presented to the ER one week later with sudden left eye vision loss and the ophthalmologic exam showed iritis, scleritis, and posterior uveitis, which was against GCA. A prominent orange-red macular rash also developed on the palms and soles of the patient, giving rise to suspicion of tertiary syphilis with ocular manifestation. His labs were unremarkable except for serum and CSF RPR/VDRL (Rapid Plasma Reagin/Venereal Disease Research Laboratory), which were strongly positive. The patient was allergic to penicillin, so he was transferred to the medical intensive care unit (MICU) for desensitisation and was started on penicillin G and probenecid. The therapy was tolerated well by the patient and he was discharged on 2.4 million unit’s penicillin G intramuscular (IM) in two divided doses daily with probenecid 500 mg every six hours for 10 days, as he was not willing to stay. Ten days later the patient was followed up on call and he stated that his symptoms had markedly improved over the course of the therapy.

## Discussion

Syphilis is generally acquired by close sexual contact but may also be acquired by non-sexual direct contact and by an unborn fetus from an infected mother. *Treponema pallidium* is a spirochete bacterium that causes syphilis, it enters the host via breaches in the squamous or columnar epithelium. There is an increase in the frequency of cases among homosexuals as compared to heterosexuals [7]. HIV-infected individuals are more likely to have contracted syphilis as compared to HIV uninfected individuals.

Ocular syphilis can occur at any time during the course of syphilis but usually accompanies early neurosyphilis with acute meningitis [8]. The predictor for neurosyphilis is an RPR titre >1:32 [9]. Ocular syphilis has many manifestations, predominately in secondary and tertiary syphilis. the most common manifestations are posterior uveitis accompanied by interstitial keratitis, chorioretinitis, neuroretinitis, and similar disorders.

Penicillin is the recommended treatment for syphilis and may also reverse retinal changes and restore visual acuity [10]. The patient initially complains of blurry vision but if treatment is not provided on time, it can lead to permanent visual loss.

## Conclusions

The spirochete Treponema Pallidum causes syphilis infection. A rare manifestation of syphilis is ocular neurosyphilis and it can imitate the presentation of autoimmune diseases like giant cell arteritis because it also presents with sudden vision loss and headache.

We want to notify physicians to consider the diagnosis of ocular syphilis in homosexual men who present with sudden vision loss, along with other routine differential diagnoses. Ocular syphilis is usually diagnosed late and may induce irreversible vision loss.

## References

[1] Kojima N, Klausner JD (2018). An update on the global epidemiology of syphilis. Curr Epidemiol Rep.

[2] Lafond RE, Lukehart SA (2006). Biological basis for syphilis. Clin Microbiol Rev.

[3] Ghanem KG, Ram S, Rice PA (2020). The modern epidemic of syphilis. N Engl J Med.

[4] (2021). EyeWiki-Ophthalmologic Manifestations of Syphilis. https://eyewiki.aao.org/Ophthalmologic_Manifestations_of_Syphilis.

[5] Hayreh SS, Podhajsky PA, Zimmerman B (1998). Ocular manifestations of giant cell arteritis. Am J Ophthalmol.

[6] Francisco JDW MD; Dean Eliott, MD; J. Michael Jumper, MD; Emmett T. Cunningham Jr, MD MD, PhD PhD, MPH MPH, Los Angeles/San (2008). How to Recognize Ocular Syphilis. https://www.reviewofophthalmology.com/article/how-to-recognize-ocular-syphilis.

[7] Read P, Fairley CK, Chow EP (2015). Increasing trends of syphilis among men who have sex with men in high income countries. Sex Health.

[8] Paulraj S, Ashok Kumar P, Gambhir HS (2020). Eyes as the window to syphilis: a rare case of ocular syphilis as the initial presentation of syphilis. Cureus.

[9] Henry K, Harwell JI (2022). Neurosyphilis: how do you know, and what do you do?. AIDS Clin Care.

[10] Whitlow CB (2004). Bacterial sexually transmitted diseases. Clin Colon Rectal Surg.

